# Office Visitors

**Published:** 1907-01

**Authors:** 


### OFFICE VISITORS.

He was a bustling, busy man and had gotten to that point in life when he found that it would be to his benefit and satisfaction to take unto himself a second wife, to fill the place of his lost first one, whom he had mourned fully the time marked by society as the proper thing.

"Doctor, I'm in a devil of a fix. I've addressed a splendid widow and she has accepted me on condition, and that is that I have these old teeth extracted and good-looking false ones inserted. Now I'm more than willing if the job could be accomplished. You see, I'd have had it done long ago, but the minute you put anything to the roof of my mouth immediately I

try to turn wrong side outwards and expectorate my boot heels. But I want the charming widow. O! but she is fine, and I must try to comply with the agreement, if I don't eat anything in a week and cavort like a wind bellows when you try the impression stunt.''

He took his seat in the chair and with great fortitude and with the help of anesthetics bore the ordeal of the extraction of the broken-down teeth pretty well. In proper time he reappeared for the taking of the impressions. Well, it was the most obstinate case of hypersensitiveness of the mouth we ever had. Great guns, how he retched and of a sudden his mouth became a spraying apparatus and blew the soft plaster out and bespattered our front windows as though he was a patent whitewashing machine. He was full of grit and at last after several efforts we did get something of an impression in waxy material—holding to him grimly as he twisted, turned, upheaved and blew. The sets were made and inserted, but what a doleful tale he would bring, as he daily appeared at our office for a month, of how he was starved, for if he tried to wear them five hours even after a meal, he would have a spasm! He actually lost flesh and rapidly became almost emaciated, but gradually became accustomed to the teeth. It was several months after his last visit before we saw him, when he marched into our office with a fine, stately woman on his arm.

''I've got the widow at last, Doctor. Ain't she fine? And I've come to acquaint you with my prize and to thank you for helping me win her.''

Of course we ordered up champagne.

